# Label-free detection of myocardial ischaemia in the perfused rat heart by spontaneous Raman spectroscopy

**DOI:** 10.1038/srep42401

**Published:** 2017-02-10

**Authors:** Suguru Ohira, Hideo Tanaka, Yoshinori Harada, Takeo Minamikawa, Yasuaki Kumamoto, Satoaki Matoba, Hitoshi Yaku, Tetsuro Takamatsu

**Affiliations:** 1 Department of Pathology and Cell Regulation, Kyoto Prefectural University of Medicine, Graduate School of Medical Science, 465 Kajii-cho Kawaramachi-Hirokoji, Kamigyo-Ku, Kyoto 602-8566, Japan; 2 Department of Cardiovascular Surgery, Kyoto Prefectural University of Medicine, Graduate School of Medical Science, 465 Kajii-cho Kawaramachi-Hirokoji, Kamigyo-Ku, Kyoto 602-8566, Japan; 3 Tokushima University, Graduate School of Science and Technology, 2-1, Minami-Josanjima, Tokushima 770-8506, Japan; 4 Department of Cardiovascular Medicine, Kyoto Prefectural University of Medicine, Graduate School of Medical Science, 465 Kajii-cho Kawaramachi-Hirokoji, Kamigyo-Ku, Kyoto 602-8566, Japan; 5 Department of Medical Photonics, Kyoto Prefectural University of Medicine, 465 Kajii-cho Kawaramachi-Hirokoji, Kamigyo-Ku, Kyoto 602-8566, Japan

## Abstract

Raman spectroscopy, which identifies intrinsic molecular constituents, has a potential for determining myocardial viability under label-free conditions. However, its suitability for evaluating myocardial ischaemia is undetermined. Focusing on cytochromes, i.e., representative molecules reflecting mitochondrial activity, we tested whether Raman spectroscopy is applicable for evaluating myocardial ischaemia especially during early ischaemic phase. We obtained spontaneous Raman spectra of the subepicardial myocardium in the Langendorff-perfused rat heart upon 532-nm excitation before and during the “stopped-flow,” global ischaemia. Semi-quantitative values of the peak intensities at 750 and 1127 cm^−1^, which reflect reduced cytochromes c and b, increased immediately and progressively after induction of the stopped flow, indicating progressive reduction of the mitochondrial respiration. Such spectral changes emerged before the loss of 1) mitochondrial membrane potentials measured by the fluorescence intensity of tetramethyl rhodamine ethyl ester or 2) staining of the triphenyl tetrazolium chloride dye in the myocardium. The progressive increases in the Raman peaks by stopped flow were significantly retarded by ischaemic preconditioning. Sequential measurements of the peak intensities at 750 and 1127 cm^−1^ enabled early detection of the myocardial ischaemia based on the mitochondrial functions. These data suggest that Raman spectroscopy offers the potential to evaluate acute ischaemic heart under label-free conditions.

Impairment of the coronary blood supply causes myocardial ischaemia, which progressively attenuates the contractile functions and viability of cardiomyocytes^1^,^2^. Under ischaemic conditions, interruption of mitochondrial respiration increases the amounts of the reduced forms of cytochromes in mitochondria^3^,^4^,^5^,^6^, and further prolongation of ischaemia leads to a loss of the mitochondrial membrane potentials, resulting eventually in cell death^7^,^8^. Although the myocardial viability is conventionally evaluated via mitochondrial activity by macroscopic stainability of triphenyl tetrazolium chloride (TTC)^9^ or tetramethyl rhodamine ethyl ester (TMRE) fluorescence for mitochondrial membrane potential^7^, it remains to be determined whether and to what extent the myocardium is under ischaemic conditions, especially during early, reversible conditions.

Spontaneous Raman microscopy provides quantitative information on substances according to their intrinsic, molecule-specific vibrational signatures of chemical bonds as a spectrum, which exhibits sharp spectral features based on specific molecular structures and conformations of tissues^10^,^11^,^12^,^13^. Recently, spontaneous Raman spectroscopy has been applied to evaluate various biological tissues or cells without chemical labeling^10^,^11^,^12^. For example, it can be used for cell imaging in the living organism^10^, and for discrimination of specific molecules in normal^12^ or diseased tissues^13^,^14^. In this regard, spontaneous Raman spectroscopy has potential for evaluation of ischaemic conditions of the heart. We previously demonstrated that the spontaneous Raman spectra of the myocardium are derived mainly from cytochromes, i.e., heme-proteins that are oxidized during respiration of the mitochondria^13^,^14^. Subsequently, Brazhe *et al*.^15^ further demonstrated the availability of Raman spectroscopy for monitoring redox states of the heart via cytochromes and myoglobin in the perfused rat heart under stopped-flow ischaemia and subsequent reperfusion. However, unknown is availability of Raman spectroscopy in comparison with other modalities for evaluating myocardial ischaemia, especially during nascent conditions of the reversible ischaemia. In this study, we have hypothesized that myocardial ischaemia can be evaluated by Raman spectroscopy in accordance with the progressive alterations of redox status of the mitochondria-derived cytochromes from the early, reversible stage to the late, irreversible one. To address this possibility, we conducted sequential detections of the Raman spectra of Langendorff-perfused rat hearts under “stopped-flow”, global ischaemia. We also evaluated applicability of the Raman spectral changes for mitochondrial functional status of the myocardium by comparison with TMRE fluorescence-based mitochondrial membrane potentials, TTC dye stainability for mitochondrial activity, and oxygen consumption rates. Applicability of Raman spectroscopy to ischaemic myocardium was also evaluated by ischaemic preconditioning^16^.

## Results

### Raman spectra

**Figure 1:**
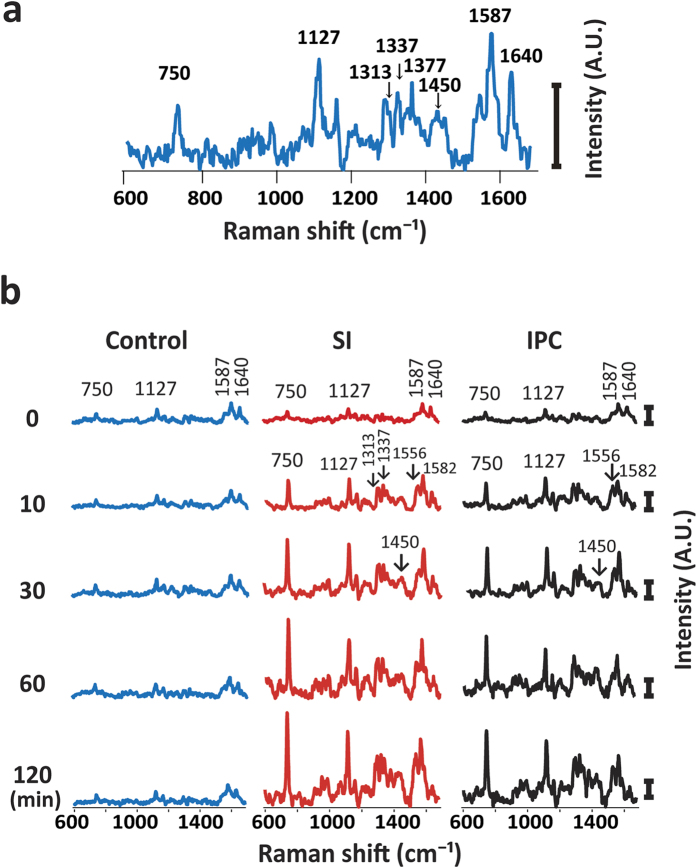
(**a**) A Raman spectrum of the perfused rat heart. It has mainly two strong peaks at 1587 and 1640 cm^−1^, and less intensive peaks at 750 and 1127 cm^−1^. (**b**) Raman spectral traces of perfused rat hearts under control, global ischaemia with ischaemic preconditioning (IPC) and without IPC (SI). The left uppermost trace is shown as a compressed form of that in (**a**). After global ischaemia, the intensity at 750 cm^−1^ was increased. Control (n = 3), SI group (n = 8), and IPC group (n = 9). Note that the scale bars in (**b**) are all the same.

**Figure 2:**
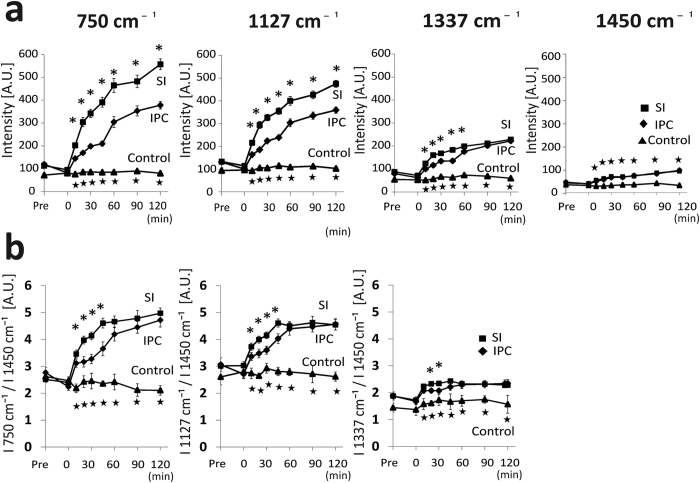
The temporal changes of the Raman spectral peak intensities. **(a)** Graphs plotting absolute peak values at 750 cm^−1^, 1127 cm^−1^, 1337 cm^−1^, and 1450 cm^−1^. **(b)** Graphs plotting ratio of the peak values at 750 cm^−1^, 1127 cm^−1^, and 1337 cm^−1^ over the peak value at 1450 cm^−1^. Each group corresponds to that in Fig. 1. Asterisks denote P < 0.05 by nonparametric Kruskal-Wallis test between each group and the control corresponding to the same time. Control (n = 3), SI group (n = 8), and IPC group (n = 9). IPC = ischaemic preconditioning; SI = simple ischaemia.

The Raman spectra of the perfused hearts had two strong peaks at 1587 and 1640 cm^−1^, and less intensive peaks at 750 and 1127 cm^−1^ (Fig. 1a). According to the previous reports^10^,^13^,^15^,^17^, the two strong peaks at 1587 and 1640 cm^−1^ correspond to the spectra of oxygenated myoglobin (oxy-Mb), and the two weak peaks at 750 and 1127 cm^−1^, reduced cytochrome c and cytochrome b. The peak at 1313 cm^−1^ is specific for cytochrome c, while that of 1337 cm^−1^ is specific for cytochrome b. The weak peak at 1377 cm^−1^ corresponds to that of oxy-Mb. Prolonged perfusion of the heart with blood-free solution showed no significant changes in the Raman spectra up to 120 min. The redox states of cytochrome c and b, and oxygenated state of Mb were stable even under long-term perfusion of the blood-free solution: we observed no discernible changes in the four peaks at 750, 1127, 1587, and 1640 cm^−1^ up to 180-min perfusion (Fig. 1b). After an induction of global ischaemia (SI) by stopped-flow, the Raman spectral peaks showed a progressive increase in intensity at 750 and 1127 cm^−1^, while those remained stable under continuous perfusion (Fig. 2a). In addition, ischaemic preconditioning (IPC) significantly reduced the progressive increase in these peak intensities that were induced by global ischaemia. The peak at 1337 cm^−1^, which is assigned to cytochrome b, was also augmented by stopped-flow, albeit weaker peak than those at 750 and 1127 cm^−1^ with or without IPC (Fig. 2a). The peak absolute values of these 3 wave numbers, however, showed no steady state by stopped flow, which may include certain progressive “background” other than the genuinely assigned components related to ischaemia despite subtraction of the background. In contrast, the Raman peak at 1450 cm^−1^, which are reportedly assigned to CH_2_ bending for lipids^18^ and stable in amount in the heart during early ischaemia^19^, showed relatively small increments with time. We therefore plotted the sequential changes in the relative peak values at 750, 1127, and 1337 cm^−1^ over the corresponding peak amplitudes at 1450 cm^−1^ (Fig. 2b). Despite the smaller changes in the ratios as compared with those in the absolute values, the ratios of these peaks at 750, 1127, and 1337 cm^−1^ over those at 1450 cm^−1^ still showed quick increments with time. In addition, the relative values for these three peaks appeared to reach quasi-steady states by 60-min stopped flow. These relative value plots also indicate that ischaemic preconditioning retards the early increments in the reduced cytochromes after the stopped flow. For the spectral peak at 1640 cm^−1^, the absolute peak intensity seemed slightly increased due possibly to the increase in the background intensity; however, the relative values were not significantly changed after stopped flow when the peak values were divided by those at 1450 cm^−1^, but were progressively decreased when divided by the whole Raman spectral values (Supplementary Fig. S1). In contrast to these intrinsic peaks, two peaks were developed at 1556 and 1582 cm^−1^ by stopped-flow, corresponding to deoxy-Mb and reduced cytochromes, respectively (Fig. 1).

### Oxygen consumption measurement

**Figure 3:**
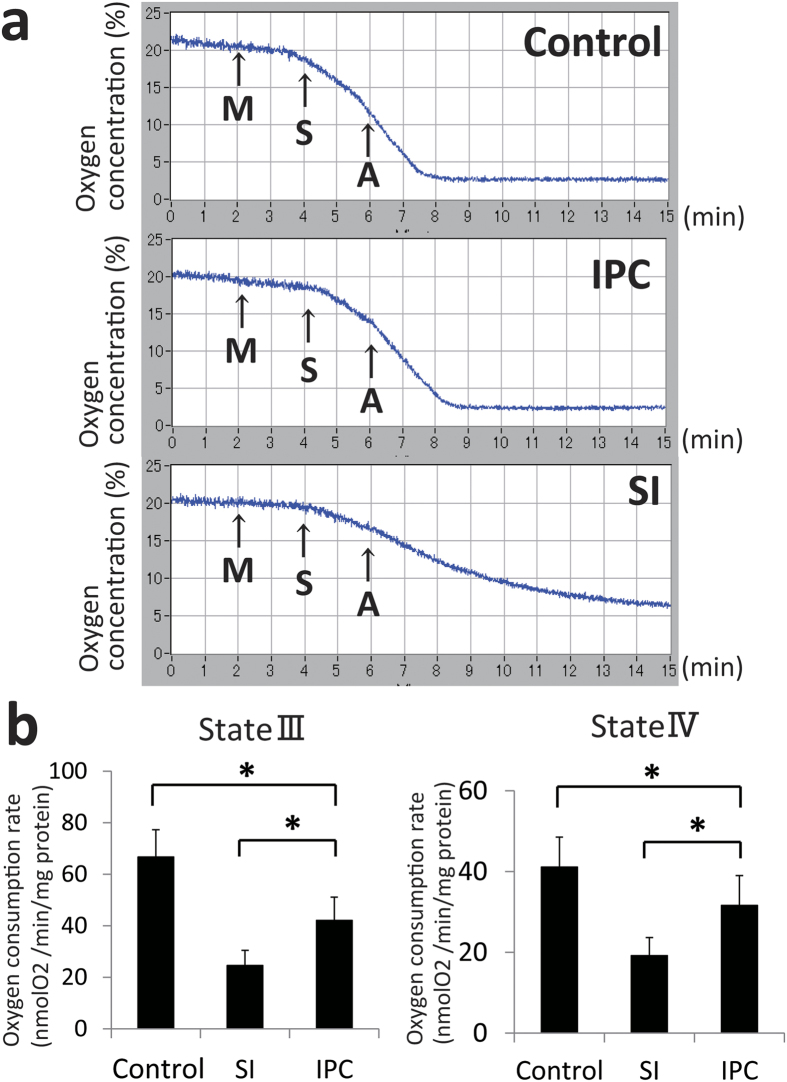
Oxygen consumption measurement after 20-min ischaemia. (**a**) The sequential state III and state IV oxygen consumption. A = Adenosine diphosphate, 1 mM; IPC = ischaemic preconditioning; SI = simple ischaemia; M = Mitochondria, 750 μg; S = Succinate, 5 mM. (**b**) Comparison of oxygen consumption calculated by the slope of oxygen consumption curve. IPC significantly reduced the decrease of oxygen consumption compared to hearts without IPC in both state III and state IV (*indicates P < 0.05). Control (n = 4), SI group (n = 4), and IPC group (n = 5).

We confirmed that the oxygen consumption was significantly decreased during ischaemia (Fig. 3a). In addition, the decrease of the oxygen consumption rate after 20-min of ischaemia was larger in the SI group than that of in the IPC group in both state III (control: 66.7 ± 10.6 vs SI: 24.6 ± 5.9 vs IPC: 42.1 ± 9.0 nmol O_2_/min/mg/protein, P < 0.05) and state IV (control: 44.1 ± 7.4, SI: 19.2 ± 4.5, and IPC: 31.6 ± 7.4 nmol O_2_/min/mg/protein, P < 0.05) (Fig. 3b).

### Mitochondrial membrane potential and histochemical evaluation

**Figure 4:**
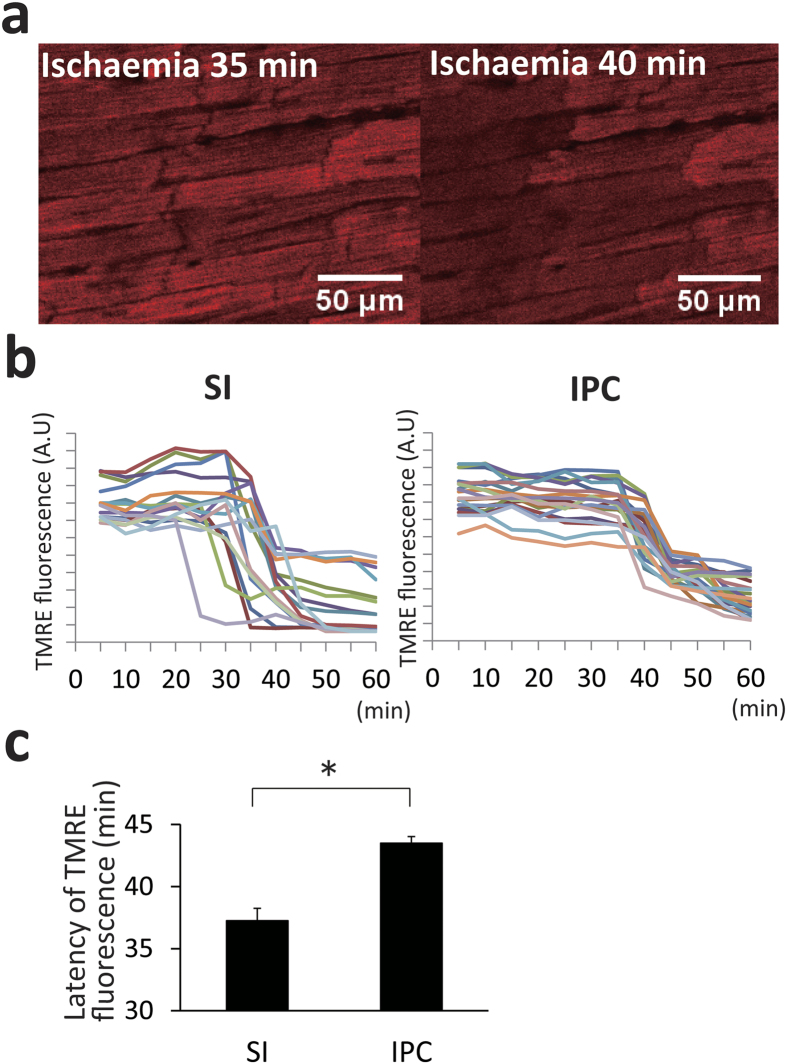
Time-lapse images of the cardiomyocytes for evaluation of the mitochondrial membrane potential indicated with tetramethyl rhodamine ethyl ester (TMRE). Images were taken every 5 min after ischaemia. A total of 6 hearts were analyzed: SI group (n = 3), and IPC group (n = 3). (**a**) Representative images of the heart. (left) 35 minutes, and (right) 40 minutes after ischaemia. (**b**) Time courses of TMRE fluorescence after ischaemia of each cardiomyocyte (n = 20, respectively). (**c**) Comparison of the latency of TMRE loss. IPC prolonged the latency as compared with the heart without IPC (43.5 min vs. 37.2 min, P < 0.05). IPC = ischaemic preconditioning; SI = simple ischaemia.

In contrast to the relatively quick, progressive changes in the Raman spectra by stopped-flow ischaemia, the mitochondrial membrane potentials were resistant to being suppressed by stopped-flow. Representative confocal images of TMRE fluorescence showed a remarkable loss of intensity after stopped-flow in the individual myocardial cells (Fig. 4a). During the global ischaemia, the mitochondrial membrane potentials were not decreased earlier than 20 min after commencement of the stopped-flow (Fig. 4b). The latency of TMRE fluorescence loss was significantly prolonged by the IPC as compared with SI (SI: 37.3 ± 1.0 vs IPC: 43.5 ± 0.5 min, P < 0.01, Fig. 4c).

As compared with the Raman spectra, TTC staining of the heart also lagged in showing the ischaemic injury. After 120 min stopped-flow, the myocardium showed significant increase in the TTC bleached area (Supplementary Fig. S2a), and significant reduction of the area as compared with SI (control: 3.9 ± 6.0% vs SI: 74.3 ± 6.4% vs IPC: 56.7 ± 7.1%, P < 0.05). However, the ischaemic injury was not detected by TTC after the 30-min stopped flow ischaemia; the myocardium barely showed bleached area at this time point (Fig. S2b). Morphologically, no discernible change was observed in the H & E images and membrane dye di-4-ANEPPS fluorescence images after 30 minute of ischaemia (Supplementary Figs S3 and S4). Thus, spontaneous Raman spectroscopy is useful for detecting the myocardial ischaemia which arises earlier before the emergence of the changes in the mitochondrial membrane potential fluorescence, TTC stainability, and morphological changes.

## Discussion

The present study was aimed to evaluate applicability of spontaneous Raman spectroscopy for detection of myocardial ischaemia in the perfused rat heart. During the early period of ischaemia, we confirmed that morphological changes are absent or too subtle to be recognized in the myocardium in the H & E histology and di-4-ANEPPS fluorescence images reflecting the cell membrane integrities. In addition, the histochemical evaluations of myocardial injury by TTC staining, which have been accepted to detect early ischaemic injury, were also difficult to be recognized as compared with the Raman spectroscopy. The TMRE fluorescence, which reflects the mitochondrial membrane potentials with high sensitivity for evaluating the irreversible mitochondrial injury, still failed to detect the early (within 30 min) ischaemic changes.

By focusing on cytochromes, we found that spontaneous Raman spectroscopy is applicable to evaluate ischaemic myocardium in an early phase. It was found that Raman-spectral peak intensities at 750 or 1127 cm^−1^ were increased within 10 min, which is significantly earlier than the changes detected by other modalities such as the TMRE fluorescence, TTC stain, or conventional histology. Previously, application of the spontaneous Raman spectroscopy to the whole heart was performed by Brazhe *et al*.^15^, who demonstrated changes in the redox states of cytochromes by stopped-flow ischaemia and reperfusion. In the present study, we performed precise sequential analyses in the Raman spectra that reflect functional status of the mitochondria especially from the early ischaemic, to advanced, irreversibly damaged conditions. The perfused rat heart showed four strong Raman bands at 750, 1127, 1587, and 1640 cm^−1^ on 532-nm excitation^10^,^13^,^15^,^17^,^20^,^21^. Of these, the Raman spectra of 750 and 1127 cm^−1^ correspond to both the reduced cytochrome c and cytochrome b^13^,^21^. Since the oxidized cytochromes make much less contribution than those of the reduced ones to these two spectral peaks, the effect of oxidized cytochromes on the observed Raman spectra is considered negligible^10^,^13^,^20^,^21^. During oxidative phosphorylation in the intact mitochondria, in which cytochrome b is oxidized as a component of complex III, reduced cytochrome c is oxidized by cytochrome oxidase as a last step of electron transport with concomitant oxygen consumption^22^. In accordance with this, inhibition of mitochondrial oxidization by ischaemia eventually increases a fraction of both reduced cytochrome c and cytochrome b^3^. Further discontinuation of mitochondrial respiration results in loss of the membrane potential, leading to tissue injury and cell death as evidenced in the present study^7^,^10^,^23^.

Measuring redox state of coenzymes such as nicotinamide adenine dinucleotide (NADH) and flavin adenine dinucleotide (FAD), both of which are located at the upstream of complex III and cytochrome oxidase, has been used to evaluate ischaemia in various organs^24^. Although measurement of increased intensities of NADH and FAD autofluorescence is a valuable modality to evaluate the very early period of ischaemia, it is difficult to assess the degree of ischaemia or damage because the intensities of NADH and FAD autofluorescence reach the plateau in seconds^25^. In addition, NADH and FAD are diminished after cell death, whereas reduced cytochromes could be confirmed even in the infarcted myocardium as we have shown in previous studies^13^,^14^.

Our data demonstrated that a progressive increment of the Raman spectral peaks at 750 and 1127 cm^−1^ reflects an increase in a fraction of reduced cytochromes after ischaemia. Such spectral changes would reach a plateau when the overall cytochromes converge to a reduced form due to prolonged ischaemia. In the long run, the peaks would in turn decrease when the myocardium changed to show total ischaemic necrosis. In practice, previous studies from our laboratory demonstrated that the Raman peaks for the reduced cytochromes decline in the course of acute myocardial infarction and its repair process (up to 21 days after infarct) because the myocardial tissue is replaced by necrotic, granulation, and fibrotic tissues^14^.

The analytical usefulness of the spontaneous Raman spectroscopy was also found to be valid for acute ischaemia even when the ischaemic damage is mitigated by preconditioning. Although we have not examined the reperfusion effects on the heart after ischaemic preconditioning, a protective effect of the preconditioning on early ischaemic myocardial injury, as was indicated by retardation of the increment in reduced cytochromes, would indicate availability of Raman spectroscopy. Therefore, the spontaneous Raman spectroscopy is of great utility not only for an early detection of the myocardial ischaemia but also for a semi-quantitative assessment of the ischaemic damage on the basis of the mitochondrial functions. Given that the spontaneous Raman spectroscopy provides minimally invasive, real-time information without chemical labeling, it can potentially be applicable to examine the viability of the myocardial tissue via functional status of mitochondria in the ischaemic heart.

We should note several limitations of this study. Firstly, the spectral changes might not faithfully represent the overall ischaemic conditions of the heart since Raman spectra were taken from the subepicardial myocardium of the left ventricle. Secondly, simulated ischaemia model by stopped-flow is distinct from myocardial ischaemia *in vivo* because of the absence of the blood perfusion or mechanical contraction of the heart. Finally, we are unable to definitely detect the point of “irreversible damage” by Raman spectra despite the relatively high-sensitivity, mitochondrial state-dependent changes in the spectral peaks. Despite these limitations, spontaneous Raman spectroscopy would provide insight to direct functional assessment in an ischaemic, and possibly failing heart.

In conclusion, our present study demonstrated applicability of spontaneous Raman spectroscopy to the evaluation of ischaemic conditions of the heart based on the mitochondrial function under label-free conditions. These approaches would be useful for early detection of ischaemic injury in the whole heart.

## Materials and Methods

### Sample preparation

All animal experiments were conducted with the approval of and in accordance with guidelines from the Committee for Animal Research of Kyoto Prefectural University of Medicine (Guide for the Care and Use of Laboratory Animals. 8th edition. Washington (DC): National Academies Press (US); 2011). All surgical procedures were performed under general anesthesia of Wistar rats (10–12 weeks, 200–300 g) with pentobarbital sodium. The hearts were rapidly excised after injection of heparin (100 U/kg body weight) via the inferior vena cava. Retrograde perfusion was started via the aorta within 60 seconds with oxygenated Tyrode’s solution (NaCl 145 mM, KCl 5.4 mM, HEPES 10 mM, MgCl_2_ 1 mM, CaCl_2_ 1 mM, and glucose 10 mM, pH 7.4 adjusted by NaOH) at 37 °C. The heart was placed on the chamber maintained at 37 °C by Peltier control system. Electrocardiogram was recorded during experiments under consecutive right atrial pacing at 150 bpm. Global ischaemia was induced to the heart by stopped flow of perfusion. In some experiments ischaemic preconditioning (hereafter, IPC) was conducted by applying a short recurrent period of ischaemia (2-min stopped-flow and 2-min perfusion, three times) after initial perfusion for 25 min to stabilize the redox status of the hearts^7^,^16^,^26^. After completion of IPC maneuver, hearts were subsequently perfused for 25 min again (60 min in total). For simple ischaemia without IPC (SI), the heart was perfused for 60 min in advance before induction of the global ischaemia.

### Mitochondria isolation

After 20-min global ischaemia, hearts were rapidly minced in ice cold MES buffer [220 mM mannitol, 70 mM sucrose, 2 mM EGTA, 5 mM MOPS (pH 7.4), 2 mM taurine, and 0.2% BSA]. Heart tissue was homogenized in MES buffer with a polytron type tissue grinder followed by two quick strokes at 500 rpm with a loose fit Potter-Elvenhjem tissue grinder. The homogenate was centrifuged at 500 gr for 5 min saving the supernatant. Mitochondrial pellet, obtained from the supernatant after centrifugation at 3,000 g, was rinsed with 100 μL incubation medium (220 mM mannitol, 70 mM sucrose, 1 mM EGTA, 2 mM taurine, 10 mM MgCl_2_, 5 mM KH_2_PO_4_ and 5 mM MOPS with 0.2% BSA added, pH 7.4 adjusted by NaOH). The isolated mitochondria were incubated for 15 min on wet ice. Protein concentration was determined with BSA as a standard by a Bradford assay. All work was performed on wet ice.

### Measurement of Raman spectra

Raman spectra were obtained with a laser Raman confocal microscope [RAMAN-11 (Nanophoton, Osaka, Japan)] that has been described previously^13^,^14^. A frequency-doubled Nd:YAG laser operating at 532 nm was employed for excitation. The subepicardial heart tissue was illuminated with the point laser beam through an objective lens [UPLSAPO 10×, NA = 0.30 (Olympus, Tokyo, Japan)], and Raman spectra were obtained with a thermoelectrically cooled CCD camera [Pixis 400BR, 400 × 1340 pixels (Princeton Instruments, Trenton, NJ, USA)]. The entrance slit width of the spectrometer installed in the Raman microscope was set to 100 μm. The irradiated laser intensity at the sample plane and exposure time for each point were up to 20 mW/μm^2^ and 10 s, respectively. Raman spectra were collected at 15 and 45 min after commencement of the coronary perfusion and at 10, 20, 30, 45, 60, 90, and 120 min after the stopped-flow under conditions so as not to change the focus for measurements over the course of experiments. In total, 20 hearts were examined: control (n = 3), SI group (n = 8), and IPC group (n = 9). At each time point, spectra were collected from more than 5 points (laser measurement diameter = ca. 0.8 μm). The impact of hemoglobin on the Raman spectra was considered to be negligible because the blood was adequately washed out from the heart^14^.

### Data processing of Raman spectra

Igor Pro 6.04 software (WaveMetrics, Inc.) was utilized for preprocessing Raman spectra. We calibrated wavenumbers of all Raman spectra by using the known Raman bands of ethanol. Modified polynomial curve fitting technique was applied in order to extract the Raman spectrum from broad fluorescence background as previously reported^11^. The autofluorescence component superposed on the Raman spectrum was estimated by calculating a modified least-squares seventh-order polynomial curve with 100 iterations, and then subtracting this polynomial from the raw spectrum. Unless otherwise specified, the Raman spectral value was measured by the difference in intensity from the baseline to the peak.

### Histological evaluation

Hematoxylin and eosin (H&E) staining and TTC staining were performed 30 min and 120 min after the stopped-flow. For H&E staining the heart was fixed with 1% paraformaldehyde via coronary perfusion: control (n = 4), SI group (n = 5), and IPC group (n = 5). For TTC staining, hearts were cut into 5 slices (approx. 2-mm thickness) after the Raman spectral measurements, incubated in 1% TTC/0.1 M in phosphate-buffered solution for 30 min, and imaged by using a vital light microscope (SZX12, Olympus, Tokyo, Japan). The TTC non-stained area was considered as an infarct area. The infarct size of the left ventricle was calculated by weighted average of individual slices: control (n = 4), SI group (n = 6), and IPC group (n = 6). All images were analyzed by an image analysis software, Image J (National Institutes of Health; USA) with the threshold setting at 113/255 in 8-bit binary image.

### Oxygen consumption measurement

The sequential state IV and state III oxygen consumption rate of the isolated mitochondria (750 μg) of the rat heart was monitored with a fiber-optic system^27^. Substrates were sequentially added at final concentrations of 5 mM for succinate and 1 mM for ADP. Each slope was measured 1 min after adding the substrate for 1 min. O_2_ consumption rate (nmol O_2_/min/mg protein) was calculated by 199 (nmol) × Y/20.9 × 1000/M (μg), where M denote mitochondria (μg), and Y, slope (%/min)^28^. A total of 13 hearts were examined: control (n = 4), SI group (n = 4), and IPC group (n = 5).

### Loading of fluorescent dyes

The perfusate was equilibrated with 100% O_2_ (pH 7.4) and the temperature was maintained at 37 °C. After 15-min perfusion, the perfusate was switched to Tyrode’s solution containing the mitochondrial membrane potential indicator tetramethyl rhodamine ethyl ester (TMRE; Life Technologies) at 100 nM, and this was followed by a 15-min wash out with dye-free Tyrode’s solution^7^. Contraction-induced movement of the heart was attenuated by 2,3- butanedione monoxime (Nakalai Tesque, Japan) at 15 mM. In the IPC group, TMRE was loaded after the prior ischaemia and re-perfusion maneuver. TMRE fluorescence imaging was performed in three hearts each for the SI and the IPC group, respectively. Minute morphological changes of cardiomyocytes were observed by confocal fluorescence images of the membrane dye, Di-4-ANEPPS (50 μM; Life Technologies), loaded by 5-min perfusion of the heart under immersion in the solution (n = 3)^29^,^30^.

### Confocal microscopy and image analysis

Confocal images were obtained on the subepicardial myocardium (640 × 480 pixels, 236 × 177 μm) of the left ventricle (excitation at 543 nm and emission at 575 nm for TMRE, and excitation at 488 nm and emission at 519 nm for di-4-ANEPPS) by using an Olympus FV 1000 microscope. All images were taken every 5-min on the subepicardial myocardium of the whole heart^31^. The resulting images were digitized at an 8-bit resolution and stored as TIFF images. Image analysis was performed by Image J. Fluorescence of TMRE was measured to assess loss of mitochondrial membrane potential in an individual cell randomly selected in region of interests (5 cardiomyocytes per 1 rat heart). Latency was defined as 50% loss of TMRE fluorescence.

### Statistical analysis

Quantitative data are presented as mean ± SEM. Comparisons of continuous variables were performed with either Mann-Whitney test or Kruskal-Wallis analysis. A level of P < 0.05 was accepted as statistically significant. All statistical calculations were performed using SPSS 22.0 software (SPSS Inc, Chicago, IL, USA).

## Additional Information

**How to cite this article**: Ohira, S. *et al*. Label-free detection of myocardial ischaemia in the perfused rat heart by spontaneous Raman spectroscopy. *Sci. Rep.* **7**, 42401; doi: 10.1038/srep42401 (2017).

**Publisher's note:** Springer Nature remains neutral with regard to jurisdictional claims in published maps and institutional affiliations.

## Supplementary Material

Supplementary Information

## References

[b1] Thygesen K. . Third universal definition of myocardial infarction. Nat Rev Cardiol 9, 620–633 (2012). 22922597 10.1038/nrcardio.2012.122

[b2] Carmeliet E. Myocardial ischaemia: reversible and irreversible changes. Circulation 70, 149–151 (1984). 6327116 10.1161/01.cir.70.1.149

[b3] Lesnefsky E. J. . Ischemic injury to mitochondrial electron transport in the aging heart: damage to the iron-sulfur protein subunit of electron transport complex III. Arch Biochem Biophys 385, 117–128 (2001). 11361007 10.1006/abbi.2000.2066

[b4] Lesnefsky E. J. . Ischaemia, rather than reperfusion, inhibits respiration through cytochrome oxidase in the isolated, perfused rabbit heart: role of cardiolipin. Am J Physiol Heart Circ Physiol 287, H258–H267 (2004). 14988071 10.1152/ajpheart.00348.2003

[b5] Chandel N. S., Budinger G. R. S., Choe S. H. & Schumacker P. T. Cellular respiration during hypoxia. Role of cytochrome oxidase as the oxygen sensor in hepatocytes. J Biol Chem 272, 18808–18816 (1997). 9228055 10.1074/jbc.272.30.18808

[b6] Balny C., Anni H. & Yonetani T. A stopped-flow study of the reaction of reduced cytochrome oxidase with oxygen. J Inorg Biochem 23, 253–258 (1985). 2991464 10.1016/0162-0134(85)85032-7

[b7] Matsumoto-Ida M., Akao M., Takeda T., Kato M. & Kita T. Real-time 2-photon imaging of mitochondrial function in perfused rat hearts subjected to ischaemia/reperfusion. Circulation 114, 1497–1503 (2006). 17000908 10.1161/CIRCULATIONAHA.106.628834

[b8] Tsujimoto Y. & Shimizu S. Role of the mitochondrial membrane permeability transition in cell death. Apoptosis 12, 835–840 (2007). 17136322 10.1007/s10495-006-0525-7

[b9] Vivaldi M. T., Kloner R. A. & Schoen F. J. Triphenyltetrazolium staining of irreversible ischaemic injury following coronary artery occlusion in rats. Am J Pathol 121, 522–530 (1985). 2416222 PMC1887916

[b10] Okada M. . Label-free Raman observation of cytochrome c dynamics during apoptosis. Proc Natl Acad Sci USA 109, 28–32 (2012). 22184220 10.1073/pnas.1107524108 PMC3252932

[b11] Minamikawa T., Harada Y. & Takamatsu T. *Ex vivo* peripheral nerve detection of rats by spontaneous Raman spectroscopy. Sci Rep 5, 17165 (2015). 26602842 10.1038/srep17165 PMC4658536

[b12] Harada Y. . Intracellular dynamics of topoisomerase I inhibitor, CPT-11, by slit-scanning confocal Raman microscopy. Histochem Cell Biol 132, 39–46 (2009). 19365636 10.1007/s00418-009-0594-0

[b13] Ogawa M. . Label-free biochemical imaging of heart tissue with high-speed spontaneous Raman microscopy. Biochem Biophys Res Commun 382, 370–374 (2009). 19285035 10.1016/j.bbrc.2009.03.028

[b14] Nishiki-Muranishi N. . Label-free evaluation of myocardial infarction and its repair by spontaneous Raman spectroscopy. Anal Chem 86, 6903–6910 (2014). 24914734 10.1021/ac500592y

[b15] Brazhe N. A., Treiman M., Faricelli B., Vestergaard J. H. & Sosnovtseva O. *In situ* Raman study of redox state changes of mitochondrial cytochromes in a perfused rat heart. PLOS One 8, e70488 (2013). 24009655 10.1371/journal.pone.0070488 PMC3757006

[b16] Murry C. E., Jennings R. B. & Reimer K. A. Preconditioning with ischemia: a delay of lethal cell injury in ischemic myocardium. Circulation 74, 1124–1136 (1986). 3769170 10.1161/01.cir.74.5.1124

[b17] Adar F. & Erecińska M. Resonance Raman spectra of the b- and c-type cytochromes of succinate-cytochrome c reductase. Arch Biochem Biophys 165, 570–580 (1974). 4374136 10.1016/0003-9861(74)90284-7

[b18] Palonpon A. F., Sodeoka M. & Fujita K. Molecular imaging of live cells by Raman microscopy. Curr Opin Chem Biol 17, 708–715 (2013). 23773582 10.1016/j.cbpa.2013.05.021

[b19] van Bilsen M. . Lipid alterations in isolated, working rat hearts during ischemia and reperfusion: its relation to myocardial damage. Circ Res 64, 304–314 (1989). 2783564 10.1161/01.res.64.2.304

[b20] Brazhe N. A. . Mapping of redox state of mitochondrial cytochromes in live cardiomyocytes using Raman microspectroscopy. PLOS One 7, e41990 (2012). 22957018 10.1371/journal.pone.0041990 PMC3434226

[b21] Kakita M., Kaliaperumal V. & Hamaguchi H. O. Resonance Raman quantification of the redox state of cytochromes b and c *in-vivo* and *in-vitro*. J Biophotonics 5, 20–24 (2012). 22076935 10.1002/jbio.201100087

[b22] Ow Y. P., Green D. R., Hao Z. & Mak T. W. Cytochrome c: functions beyond respiration. Nat Rev Mol Cell Biol. 9, 532–542 (2008). 18568041 10.1038/nrm2434

[b23] Hausenloy D. J., Maddock H. L., Baxter G. F. & Yellon D. M. Inhibiting mitochondrial permeability transition pore opening: a new paradigm for myocardial preconditioning? Cardiovasc Res 55, 534–543 (2002). 12160950 10.1016/s0008-6363(02)00455-8

[b24] Papayan G., Petrishchev N. & Galagudza M. Autofluorescence spectroscopy for NADH and flavoproteins redox state monitoring in the isolated rat heart subjected to ischaemia-reperfusion. Photodiagnosis Photodyn Ther 11, 400–408 (2014). 24854770 10.1016/j.pdpdt.2014.05.003

[b25] Levitt J. M. . Intrinsic fluorescence and redox changes associated with apoptosis of primary human epithelial cells. J Biomed Opt 11, 064012 (2006). 17212535 10.1117/1.2401149

[b26] Heusch G. Molecular basis of cardioprotection: signal transduction in ischaemic pre-, post-, and remote conditioning. Circ Res 116, 674–699 (2015). 25677517 10.1161/CIRCRESAHA.116.305348

[b27] Hoshino A. . Cytosolic p53 inhibits Parkin-mediated mitophagy and promotes mitochondrial dysfunction in the mouse heart. Nat Commun 4, 2308 (2013). 23917356 10.1038/ncomms3308

[b28] Matoba S. . p53 regulates mitochondrial respiration. Science 312, 1650–1653 (2006). 16728594 10.1126/science.1126863

[b29] Jiang Y., Tanaka H., Matsuyama T., Yamaoka Y. & Takamatsu T. Pacing-induced non-uniform Ca^2+^ dynamics in rat atria revealed by rapid-scanning confocal microscopy. Acta Histochem Cytochem 47, 59–65 (2014). 25221364 10.1267/ahc.14014 PMC4138402

[b30] Matsuyama T. . Intrinsic left atrial histoanatomy as the basis for reentrant excitation causing atrial fibrillation/flutter in rats. Heart Rhythm 10, 1342–1348 (2013). 23680896 10.1016/j.hrthm.2013.04.021

[b31] Davidson S. M., Yellon D. M., Murphy M. P. & Duchen M. R. Slow calcium waves and redox changes precede mitochondrial permeability transition pore opening in the intact heart during hypoxia and reoxygenation. Cardiovasc Res 93, 445–453 (2012). 22198507 10.1093/cvr/cvr349

