# Supplementary material for: Label-free detection of myocardial ischaemia in the perfused rat heart by spontaneous Raman spectroscopy
Source: Sci Rep. 2017 Feb 10;7:42401. doi: 10.1038/srep42401 (PMC5301243; doi:10.1038/srep42401)
Supplement: Supplementary Information [file srep42401-s1.pdf]

Label-free detection of myocardial ischaemia in the perfused rat heart by spontaneous Raman spectroscopy

Suguru Ohira, Hideo Tanaka, Yoshinori Harada, Takeo Minamikawa,

Yasuaki Kumamoto, Satoaki Matoba, Hitoshi Yaku & Tetsuro Takamatsu

Supplementary information

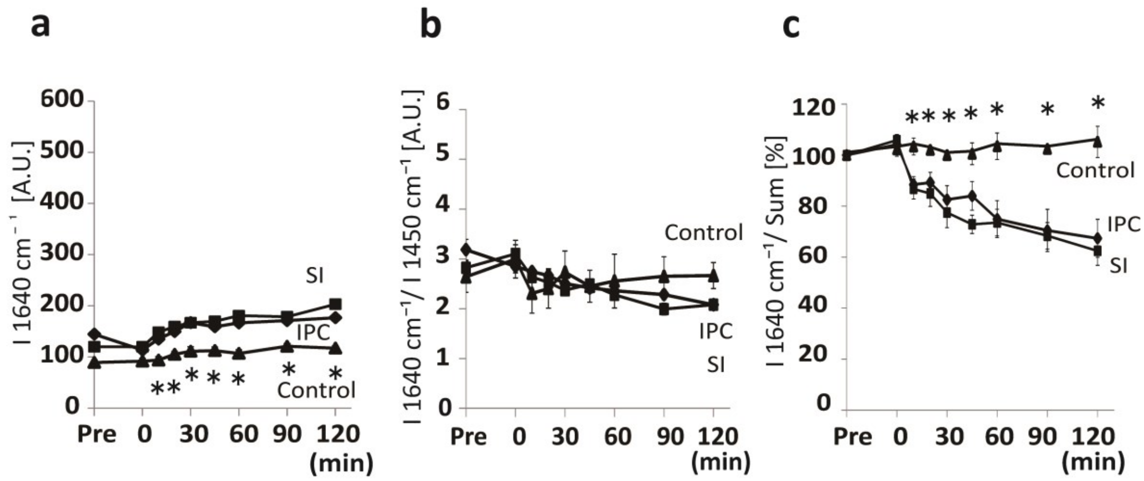

Supplementary Figure S1. The temporal changes of the Raman spectral peak intensity at 1640 cm^{-1}: absolute intensity values **(a)**, the intensity values normalized by the corresponding values at 1450 cm^{-1} **(b)**, and those divided by the whole (sum) values of the Raman spectrum **(c)**. Asterisks denote $P < 0.05$ by nonparametric Kruskal-Wallis test between each group for SI or IPC and the control corresponding to the same time. Control (n = 3), SI group (n = 8), and IPC group (n = 9). IPC = ischaemic preconditioning; SI = simple ischaemia**.**

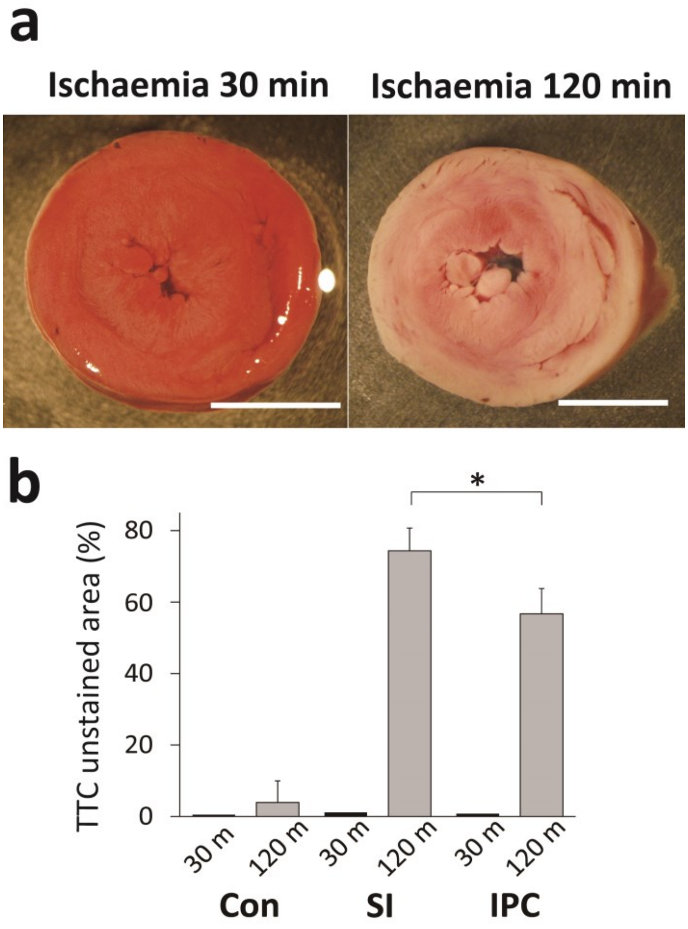

Supplementary Figure S2. Triphenyl tetrazolium chloride (TTC) staining of the heart also lagged in showing the ischaemic injury. After 120-min stopped flow, the myocardium showed significant increase in the TTC bleached area (a, right), and significant reduction of the area as compared with SI (control: 3.9 ± 6.0% vs SI: 74.3 ± 6.4% vs IPC: 56.7 ± 7.1%, P <0.05) (b). The ischaemic injury was not detected by TTC after the 30-min stopped-flow ischaemia; the myocardium barely showed bleached area at this time point (a, left). Scale bars show 5 mm. IPC = ischaemic preconditioning; SI = simple ischaemia

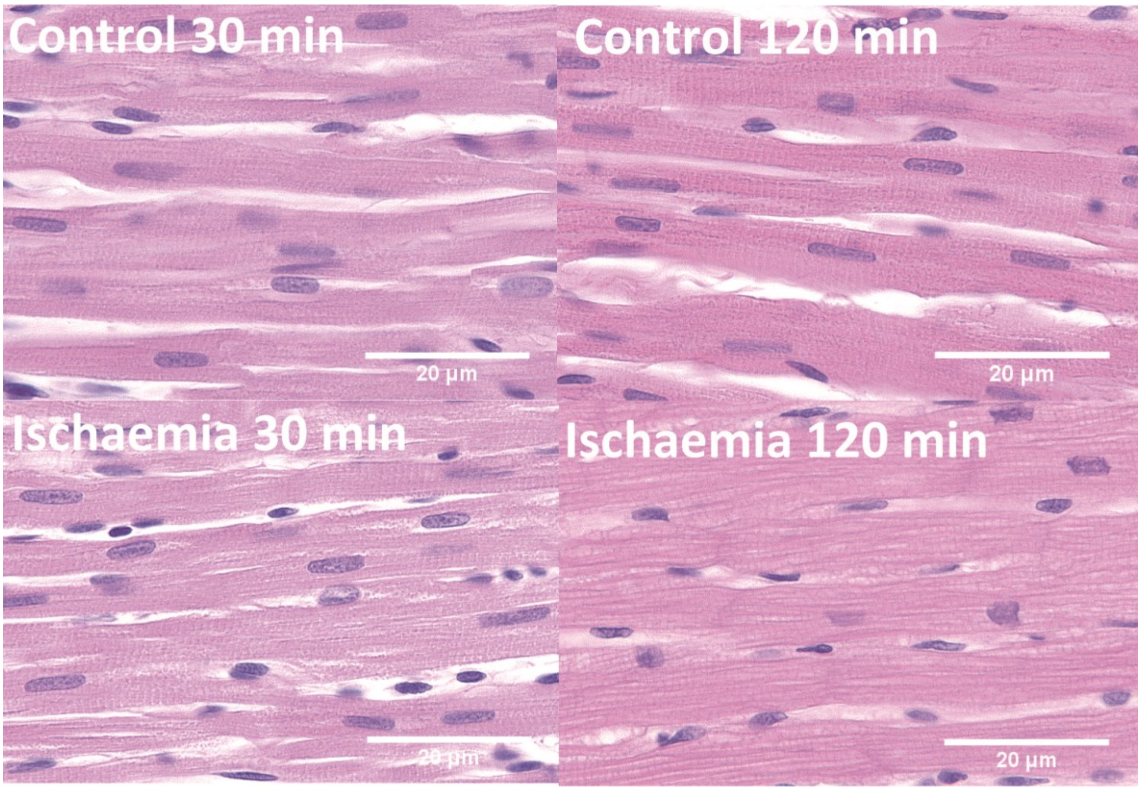

Supplementary Figure S3. H&E-stained images of the subepicardial myocardium at the anterior wall of the left ventricle. Control heart: perfusion for 30 and 120 min. Ischaemic heart: 30 min and 120 min after ischaemia. There was no obvious morphological change at 30 min of ischaemia. The cross striation was still visible in the cardiomyocytes after 120 min of ischaemia, whereas an edematous change of mitochondria was observed.

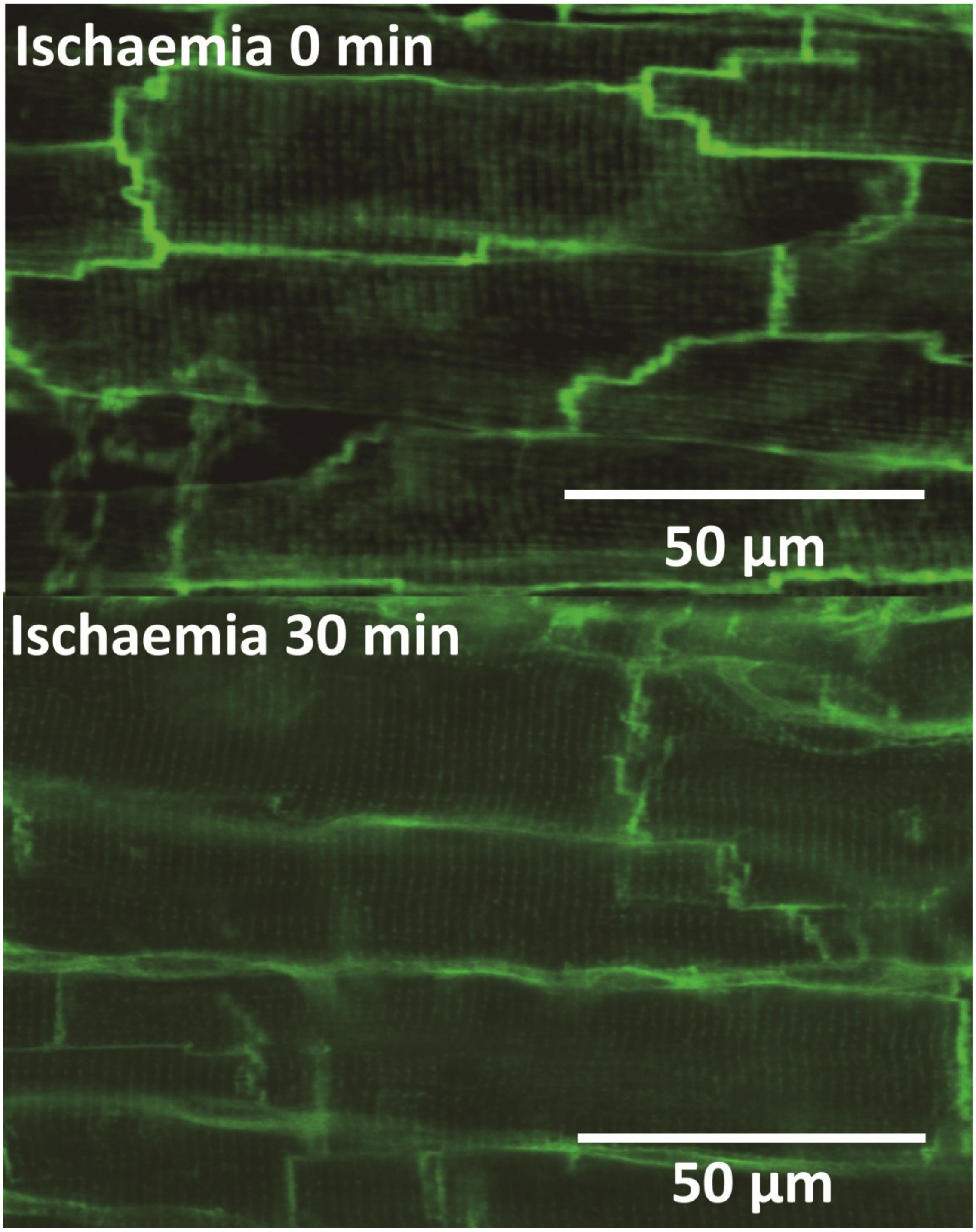

Supplementary Figure S4. Confocal images of cell structures in the anterior wall of the left ventricle in the di-4-ANEPPS-stained rat whole heart after: 0, and 30 min of ischaemia. The structure of the T-tubules in cardiomyocytes was maintained after 30 minutes of ischaemia, whereas the intercellular space became slightly edematous.
